# Application of contrast-enhanced ultrasound in the surgical treatment of vesicoureteral reflux in children

**DOI:** 10.1007/s00383-023-05605-9

**Published:** 2023-11-25

**Authors:** Zhao Lan Ye, Li Hua Zhang, Lin Zhu, Wei Ji Chen, Di Xu, Ning Lin

**Affiliations:** 1 Department of Ultrasound, Fujian Provincial Hospital, No.134, East Street, Fuzhou, Fujian China; 2 Pediatric Surgery, Fujian Provincial Hospital, No.134, East Street, Fuzhou, Fujian China

**Keywords:** Contrast-enhanced voiding urosonography, Vesicoureteral reflux, Ureterovesical reimplantation

## Abstract

**Background:**

To determine the utility of contrast-enhanced voiding urography (CeVUS) in the treatment of vesicoureteral reflux (VUR) through ureterovesical reimplantation in children.

**Methods:**

A total of 159 children with recurrent urinary tract infections were selected for CeVUS and voiding cystourethrography (VCUG) from December 2018 to December 2020, among whom 78 patients were eventually diagnosed with VUR. Overall, 60 pyelo-ureteric units (PUUs) were operated according to surgical indications. Accordingly, we determined the general clinical characteristics of all children, obtained two-dimensional ultrasound images, assessed the reflux status of children using the contrast-enhanced technique, and compared the obtained results via CeVUS and VCUG. Both imaging modalities were reperformed at 6, 12, and 18 months after surgery to evaluate postoperative outcomes. In particular, we assessed the consistency of the evaluation and calculated the diagnostic efficacy of CeVUS for different levels of reflux at different time points.

**Results:**

CeVUS showed considerable efficacy in the diagnosis of children with VUR. Notably, the diagnostic results of both CeVUS and VCUG achieved high agreement, with a kappa value of 0.966 (*P* < 0.001). The results of our follow-up at different stages and evaluation of postoperative efficacy revealed that CeVUS possessed substantial diagnostic efficacy and good consistency with VCUG.

**Conclusion:**

CeVUS is an accurate and safe examination, with considerable clinical significance for diagnosing VUR in children, determining the treatment approach, conducting follow-up during treatment, and evaluating subsequent treatment outcomes.

## Introduction

Vesicoureteral reflux (VUR) is a disorder of the urinary system commonly observed in children. It is an abnormality of the antireflux function or structure of the vesicoureteral junction, which is caused by various factors, resulting in urine flow into the ureter, renal pelvis, and renal sinus [1]. Studies have reported VUR incidence rates of 1–2% [2], which have reached as high as 30–50% in patients with urinary tract infections (UTIs) and fever [3]. As the severity of VUR reflux increases, UTI remission rates tend to decrease, whereas recurrence rates tend to increase [4]; only children with mild reflux could experience self-healing. Moreover, a study suggested that higher degrees of reflux can lead to more serious consequences, such as intrarenal hypertension, renal insufficiency, and eventually renal scar formation [4]. Thus, an accurate diagnosis and effective surgical intervention in the early stage of high-grade VUR in children are particularly important to reduce subsequent renal damage and protect renal function. Although some children with low-grade VUR may experience relief naturally, regular follow-up is required during this process to determine whether reflux persists. Considering the atypical and nonspecific early clinical manifestations of VUR, imaging plays a particularly important role in its diagnosis [5]. Currently, excretory cystourethrography (VCUG) is the most commonly used modality for diagnosing reflux and is the gold standard for grading it [6].

With the advent of the second-generation ultrasound contrast agent SonoVue and the clinical use of high-resolution ultrasound machines, contrast-enhanced voiding urography (CeVUS) is being increasingly used for diagnosing VUR. In Europe, CeVUS has even become a routine examination for screening children with VUR [6]. CeVUS is a dynamic imaging technology that examines the structure of the urinary tract after the administration of contrast agents into the bladder. This technology enables continuous real-time urinary tract scanning to assess the renal morphology and structure in children from multiple angles, allowing better assessment of the dynamic nature of intermittent reflux [7]. Compared with VCUG, radionuclide cystography, and other methods, CeVUS is not only more sensitive in detecting reflux in children but also more accurate in classifying its severity [8–10]. As no ionizing radiation is used, the examination can be repeated several times after surgery, which allows for close monitoring and dynamic observation.

Currently, however, CeVUS in China is still in its initial stages, has not been widely used, and has no uniform standard operating process. Moreover, only a few studies have performed continuous pre- and postoperative evaluation. Therefore, the current study aimed to evaluate the clinical application of CeVUS before and after laparoscopic ureteral bladder reimplantation in children with VUR and investigate its clinical utility, reliability, feasibility, and safety.

## Materials and methods

### Patient data and processing

A total of 159 children admitted to our hospital for repeated UTIs from December 2018 to December 2020 were selected. Inclusion criteria:All children had CeVUS and VCUG examinations. Children with VCUG results showing grade IV–V reflux on at least one side and satisfying the indications for the surgical treatment of VUR, based on the Expert Consensus on Primary Vesicoureteral Reflux in Children published by the Urology Group of the Chinese Medical Association Pediatric Surgery Branch in 2019, were treated with gas bladder laparoscopic ureteral bladder reimplantation for 60 PUUs, including 45 males and 15 females, aged 1.25–4.66 (mean, 1.89 ± 0.14) years. A single ureter was operated on in all children. CeVUS and VCUG were reperformed at 6, 12, and 18 months postoperatively. Exclusion criteria: children with a history of asthma; urticaria; severe respiratory, cardiac, and renal insufficiency; impaired consciousness; and other critically ill children and children with incomplete clinical information. Two children were excluded because they were unable to complete the examination due to catheter dislodgement caused by violent crying and struggling during CeVUS.The study was approved by the hospital ethics committee, and written informed consent was obtained from the children’s family members prior to the study.

### Inspection method

A Philip Epiq5 color Doppler diagnostic instrument equipped with C5-1 convex array probe, L12-3 high-frequency linear array probe, and contrast imaging technology was used. The ultrasound contrast agent SonoVue was used simultaneously. Before use, we injected 5 mL of sterile physiological saline into the contrast agent, shook the mixture until the freeze–dried powder was completely dissolved, and obtained 0.5 mL of microbubble suspension for further use.

The children did not need sedation and were asked to lie in the flat and/or lateral position. Two-dimensional grayscale images of the kidney, ureter, and bladder were routinely viewed, after which kidney size, parenchymal thickness, renal sinus separation, and ureteral dilation were measured, and the corresponding data were recorded. Kidney size was determined by measuring the long diameter of the coronal section in the lateral position as well as the right, left, anterior, and posterior diameters of the transverse section through the renal hilum in the supine position. The three measurements were multiplied to obtain kidney size. Parenchymal thickness was determined based on the mean values measured in the upper, middle, and lower parts of the coronal section in the lateral recumbent position. The renal sinus separation was assessed by measuring the anteroposterior diameter of the renal pelvis in the transverse section in the supine position.

An indwelling catheter was inserted under sterile conditions to empty their bladder. Notably, we connected the tee tube to one end of the catheter for connecting with the contrast agent and to the other end for connecting with the physiological saline, which was suspended approximately 60 cm above the horizontal plane of the bladder to facilitate retrograde bladder filling via gravity. We calculated the physiological filling volume of the bladder according to the Koff formula: physiological filling volume of the bladder (mL) = [age (in years) + 2] × 30 [11]. After the bladders reached 50% of the filling volume, we injected 0.5 mL of contrast agent suspension, dripped physiological saline onto the filling volume, and closed the catheter. During physiological bladder filling, enlarged images were obtained by focusing on the inner segment of the ureteral bladder wall. Subsequently, we measured the length and inner diameter of the inner segment of the bladder and determined the mean values at the near, middle, and far points. Then, we set the ultrasound machine to the dual view mode of angiography and the mechanical index to 0.10. When the entire bladder was evenly with microbubbles, we asked the children to urinate. Children who did not cooperate were made to cry. We assessed reflux in real-time by scanning the bladder, ureter, and renal pelvis, observing the images for 5–l0 min, and storing dynamic images. After using the contrast agent, we drained the contrast agent from the urinary tract and removed the catheter. Adverse reactions throughout and after the examination were then examined and recorded. Children who felt uncomfortable were taken care of promptly. All examinations were performed by the same senior physician who had experience in imaging techniques.

Echo-rich microbubbles were detected in the ureter, renal pelvis, and renal calyces, indicating retrograde urine flow suggestive of VUR. Depending on the location and extent of urinary tract involvement, they can be classified into five grades, similar to the five-grade classification system proposed by the International Committee on Reflux Research currently used for traditional VCUG [12]: grade I, reflux reaches the ureter; grade II, reflux into the ureter, renal pelvis, and renal calyces but without any dilatation; grade III, reflux with mild or moderate dilatation of the renal pelvis but no or only mild dullness of the renal calyces; grade IV, moderate dilatation of the renal pelvis or moderate distortion of the ureter, with complete disappearance of the acute angle of the renal calyces, which mostly remain papillary depressed; grade V, considerable dilatation of the renal pelvis and calyces; the ureter is greatly dilated and distorted, and most of the renal calyces lose their nipple shape.

### Criteria for evaluating efficacy

Complete remission was defined as negative contrast results. Partial mitigation was defined as a reduction in reflux grade by ≥ 1 level. Invalid result was defined as no reduction or worsening of reflux grade.

### Statistical analysis

Statistical analysis was performed using SPSS 22.0. Measurement data were expressed as mean ± standard deviation, and one-way analysis of variance was performed using t-test and χ^2^ test. The kappa test was used to evaluate the agreement between the two methods for diagnosing VUR. A *P* value of < 0.05 was considered to indicate statistical significance. Furthermore, the sensitivity, specificity, accuracy, positive predictive value, and negative predictive value of CeVUS for preoperative screening, 6-month postoperative follow-up, 12-month postoperative follow-up, 18-month postoperative follow-up, and clinical assessment of surgical efficacy were calculated using VCUG as a reference.

## Results

A comparison of the baseline data showed that the VUR group had a significantly higher proportion of males, number of children with a family history of VUR, and number of children with a history of urinary tract infection than the non-VUR group (*P* < 0.05). In addition, no statistically significant difference was noted between the two groups in terms of age, whether the renal sinus was separated during the fetal period, and whether the premature infant was born (*P* > 0.05; Table 1).

**Table 1:** Single-factor analysis of baseline data in children with and without vesicoureteral reflux

Parameter	VUR (n = 78)	Non-VUR (n = 79)	Statistics	*P*
Age	2.14 ± 1.13	2.08 ± 1.21	0.368	0.738
**Gender**			9.793	0.002
Male	57	36		
Female	21	43		
**Family history (whether parents, siblings have VUR)**			12.104	0.001
Yes	26	4		
No	52	75		
**History of urinary system infection**			58.008	<0.001
Yes	71	18		
No	7	61		
**Whether the renal sinus is separated in fetal period**			0.088	0.757
Yes	21	20		
No	57	59		
**Premature or not**			0.339	0.551
Yes	6	5		
No	72	74		

Note: Measurement data are presented as mean ± standard deviation. Statistical values are presented as t values, whereas the remaining statistical values are presented as χ^2^ values

Among the urological ultrasound parameters, the severity of renal sinus separation in the VUR group (2.12 ± 3.96 mm) was higher than that in the non-VUR group (0.91 ± 3.38 mm), although the difference between the two groups was not statistically significant (*P* > 0.05). The length of the inner segment of the ureteral wall was significantly shorter in the VUR group than in the non-VUR group, whereas the inner diameter of the inner segment of the ureteral wall was slightly wider in the VUR group than in the non-VUR group, and the difference between the two groups was statistically significant (*P* < 0.05). No significant differences in kidney volume, parenchymal thickness, and ureteral dilatation were observed between the VUR and non-VUR groups (*P* > 0.05; Table 2).

**Table 2:** Single-factor analysis of urinary ultrasound parameters

Parameter	VUR (n = 78)	Non-VUR (n = 79)	Statistics	*P*
Kidney volume(cm^3^)	65.24 ± 8.01	62.50 ± 11.23	1.914	0.063
Renal parenchymal thickness(mm)	9.22 ± 0.89	9.01 ± 0.67	0.866	0.397
Separation of renal sinus(mm)	2.12 ± 3.96	0.91 ± 3.38	1.940	0.054
**Whether the ureter is dilated**			1.047	0.304
Yes	13	8		
No	65	71		
Length of inner segment of ureter wall(mm)	4.68 ± 0.85	6.27 ± 0.38	4.128	<0.001
Inner diameter of ureteral wall(mm)	1.07 ± 0.13	0.98 ± 0.41	5.451	<0.001

Note: Measurement data are presented as mean ± standard deviation. Statistical values are presented as t values, whereas the remaining statistical values are presented as χ^2^ values

A total of 78 children with VUR (156 PUUs) were diagnosed using CeVUS, with 43 (27.56%) grade 0, 15 (9.61%) grade I, 16 (10.26%) grade II, 22 (14.10%) grade III, 30 (19.23%) grade IV, and 30 (19.23%) grade V PUUs.

The sensitivity of CeVUS for graded diagnosis was high, with individual sensitivities of 100% (40/40) for grade 0, 83.33% (15/18) for grade I, 94.12% (16/17) for grade II, 100% (21/21) for grade III, 96.77% (30/31) for grade IV, and 100% (29/29) for grade V. The specificity of CeVUS for graded diagnosis reached > 97%, with individual specificities of 97.41% (113/116) for grade 0, 100% (138/138) for grade I, 100% (139/139) for grade II, 99.26% (134/135) for grade III, 100% (125/125) for grade IV, and 99.21% (126/127) for grade V. The accuracy of CeVUS for graded diagnosis was ≥ 98%, with individual accuracies of 98.08% (153/156) for grade 0, 98.08% (153/156) for grade I, 99.36% (155/156) for grade II, 99.36% (155/156) for grade III, 99.36% (155/156) for grade IV, and 99.36% (155/156) for grade V. The positive predictive value of CeVUS reached > 93%, with the individual values of 93.02% (40/43) for grade 0, 100% (15/15) for grade I, 100% (16/16) for grade II, 95.45% (21/22) for grade III, 100% (30/30) for grade IV, and 96.67% (29/30) for grade V. The negative predictive value of CeVUS reached > 97%, with the individual values of 100% (113/113) for grade 0, 97.87% (138/141) for grade I, 99.29% (239/140) for grade II, 100% (134/134) for grade III, 99.21% (125/126) for grade IV, and 100% (126/126) for grade V. A comparison between VCUG and CeVUS showed that the two diagnostic modalities achieved high agreement, with a Kappa value of 0.966 (*P* < 0.001; Table 3).

**Table 3:** Comparison of the results of contrast-enhanced voiding urography and voiding cystourethrography in children with vesicoureteral reflux

VUCG Grade	CeVUS Grade						Total	Kappa	*P*
	0	I	II	III	IV	V			
0	40	0	0	0	0	0	40	0.966	<0.001
I	3	15	0	0	0	0	18		
II	0	0	16	1	0	0	17		
III	0	0	0	21	0	0	21		
IV	0	0	0	0	30	1	31		
V	0	0	0	0	0	29	29		
**Total**	43	15	16	22	30	30	156		

During the 6-month follow-up after surgery, CeVUS showed no reflux in 42 cases (70%), grade I in 17 cases (28.3%), and grade II in 1 case (1.7%). Further, the diagnostic sensitivity of the grading technique reached > 85%, and the diagnosis of grade 0 and grade II were completely consistent, with individual sensitivities of 100% (39/39) for grade 0, 85% (17/20) for grade I, and 100% (1/1) for grade II. The specificity of diagnosing all grades, except grade 0, reached 100%, with individual specificities of 85.71% (18/21) for grade 0, 100% (40/40) for grade I, and 100% (59/59) for grade II. The accuracy of CeVUS for graded diagnosis reached > 93%, with individual accuracies of 93.33% (56/60) for grade 0, 95% (57/60) for grade I, and 100% (60/60) for grade II. The positive predictive value of all grades, except for grade 0, reached 100%, with individual values of 92.86% (39/42) for grade 0, 100% (17/17) for grade I, and 100% (1/1) for grade II. The negative predictive value of CeVUS for graded diagnosis reached > 93%, with individual values of 100% (18/18) for grade 0, 93.02% (40/43) for grade I, and 100% (59/59) for grade II. A comparison between VCUG and CeVUS revealed that the results of both diagnostic modalities were consistent, with a kappa value of 0.889 (*P* < 0.001; Table 4).

**Table 4:** Comparison of the results of contrast-enhanced voiding urography and voiding cystourethrography in children at 6, 12, and 18 months after surgery

		CeVUS Grade				Kappa	*P*
		0	I	II	**Total**		
**6 months after operation**							
VUCGP Grade	0	39	0	0	39	0.889	<0.001
	I	3	17	0	20		
	II	0	0	1	1		
	**Total**	42	17	1	60		
**12 months after operation**							
VUCG Grade	0	51	0	0	51	0.856	<0.001
	I	2	7	0	9		
	II	0	0	0	0		
	**Total**	53	7	0	60		
**18 months after operation**							
VUCG Grade	0	53	0	0	53	0.815	<0.001
	I	2	5	0	7		
	II	0	0	0	0		
	**Total**	55	5	0	60		

At the 12-month follow-up after surgery, CeVUS diagnosed 53 cases (88.3%) as grade 0, 7 cases (11.7%) as grade I, and 0 cases (0%) as grade II. The sensitivity of CeVUS was 100% (51/51) for grade 0, 77.78% (7/9) for grade I, and 100% (0/0) for grade II. The diagnostic specificity of CeVUS was 77.78% (7/9) for grade 0, 100% (51/51) for grade I, and 100% (60/60) for grade II. The diagnostic accuracy of CeVUS for graded diagnosis reached > 96%, with individual accuracies of 96.67% (58/60) for grade 0, 96.67% (58/60) for grade I, and 100% (60/60) for grade II. The positive predictive values were 96.23% (51/53) for grade 0 and 100% (7/7) for grade (I) The negative predictive values were 100% (7/7) for grade 0, 96.23% (51/53) for grade I, and 100% (60/60) for grade (II) The results of the two diagnostic modalities were found to be consistent, with a kappa value of 0.856 (*P* < 0.001; Table 4).

At the 18-month follow-up after surgery, CeVUS diagnosed 55 cases (91.7%) as grade 0, 5 cases (8.3%) as grade I, and 0 cases (0%) as grade II. The sensitivity of CeVUS was 96.36% (53/55) for grade 0, 71.43% (5/7) for grade I, and 100% (0/0) for grade II, whereas its specificity was 71.43% (5/7) for grade 0, 100% (53/53) for grade I, and 100% (60/60) for grade II. The diagnostic accuracy of CeVUS was 96.67% (58/60) for grade 0, 96.67% (58/60) for grade I, and 100% (60/60) for grade II. The positive predictive values of CeVUS were 96.36% (53/55) for grade 0 and 100% (5/5) for grade (I) The negative predictive values of CeVUS were 100% (5/5) for grade 0, 96.36% (53/55) for grade I, and 100% (60/60) for grade (II) The diagnostic results of the two diagnostic modalities were consistent, with a kappa value of 0.815 (*P* < 0.001; Table 4).

The current study revealed that CeVUS and VCUG were highly consistent in terms of their efficacy in assessing children at all postoperative periods (6-,12-, and 18-month follow-up), with kappa values of 0.886, 0.856, and 0.815, respectively (*P* < 0.001). During efficacy assessment at 6, 12, and 18 months after surgery, the sensitivity was 100% at all time points; specificity was 85.71%, 77.78%, and 71.43%; accuracy was 95%, 96.67%, and 96.67%; positive predictive value was 92.86%, 96.23%, and 100%; and negative predictive value was 100% at all time points, respectively (Table 5).

**Table 5:** Comparison of contrast-enhanced voiding urography and voiding cystourethrography in the evaluation of postoperative efficacy

		CeVUS			Kappa	*P*
		Complete remission	Partial mitigation	Total		
**6 months after operation**					0.886	<0.001
VUCG	Complete remission	39	0	39		
	Partial mitigation	3	18	21		
	Total	42	18	60		
**12 months after operation**					0.856	<0.001
VUCG	Complete remission	51	0	51		
	Partial mitigation	2	7	9		
	Total	53	7	60		
**18 months after operation**					0.815	<0.001
VUCG	Complete remission	53	0	53		
	Partial mitigation	2	5	7		
	Total	55	5	60		

**Fig. 1:**
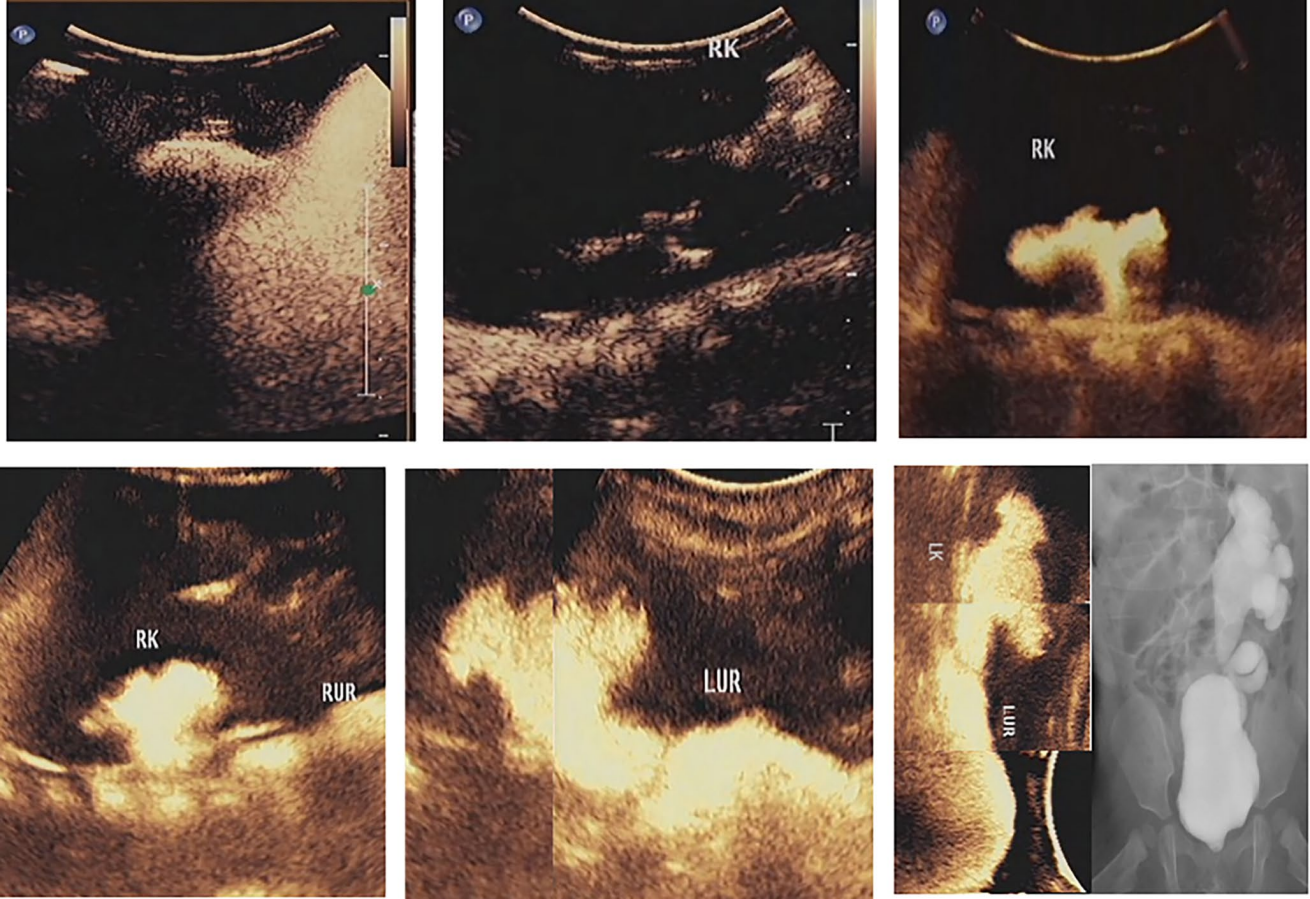
Ultrasound angiogram of vesicoureteral reflux. (Upper left: diagram of a grade I left cystoureteral reflux; upper middle: diagram of a grade II right cystoureteral reflux; upper right: diagram of a grade III right cystoureteral reflux; lower left: diagram of a grade IV right cystoureteral reflux; lower middle: diagram of a grade V left cystoureteral reflux; lower right: comparison diagram of a grade V left cystoureteral reflux detected using contrast-enhanced voiding urography and voiding cystourethrography)

CeVUS can clearly diagnose the grading of reflux in children with VUR and obtain similar morphological images of the kidney, ureter, and bladder as VCUG through multislice image capture and splicing of different parts (Fig. 1).

## Discussion

CeVUS is a safe, radiation-free, highly sensitive, specific, and accurate imaging technique that plays a critical role in different stages of VUR diagnosis and treatment.

VCUG has been the gold standard and traditional imaging method for the clinical diagnosis of VUR. Considering the greater susceptibility of children’s growing tissues to radiation, particularly in children with VUR who require long-term follow-up, than adult tissues, increase in the number of postoperative examinations even in a short period, and accumulation of ionizing radiation damage, which may show potential carcinogenic effects during their life expectancy, it is necessary to further reduce the radiation exposure of patients undergoing VCUG even in cases where intermittent fluoroscopic screening and pulsed digital fluoroscopy are used [11, 12]. A pediatric radiation dose progression study by Sulieman et al. revealed that cumulative radiation exposure injury and developmental effects of radiation on the gonads were unavoidable in children with VUR requiring repeated examinations [13, 14].

CeVUS is substantially different from VCUG as it enables physicians, children with VUR, and their families to complete the examination in a completely radiation-free manner, without any risk of radiation accumulation and harm to the growth and development of younger children, regardless of whether the examination is repeated several times within a short period. Regarding the contrast agent, SonoVue has been safely used as an intravesical injection, with extremely rare systemic complications [15]. Furthermore, a recent European-wide questionnaire survey of 5079 CeVUS examinations in children performed at 45 European centers reported no allergic reactions or systemic complications associated with SonoVue and found only a few minor complications related to catheterization [16]. Our study also confirmed the enhanced safety of CeVUS, wherein no children developed contrast-related adverse reactions, such as nausea, vomiting, abdominal pain, or catheterization- or infection-related complications.

In this study, CeVUS exhibited high sensitivity, specificity, and accuracy in diagnosing VUR. During preoperative screening, the sensitivity of CeVUS for graded diagnosis was > 83%, with a sensitivity of 100% for grades 0, III, and V. Meanwhile, its specificity exceeded 97%, with a specificity of 100% for grades I, II, and V. Moreover, the accuracy of the technique exceeded 98%, with positive and negative predictive values of > 95%. Our postoperative data showed that sensitivity and specificity of CeVUS exceeded 71%, and its accuracy, positive predictive value, and negative predictive value exceeded 92%. In the efficacy assessment, sensitivity and negative predictive value were 100%, specificity was > 71%, and accuracy and positive predictive value were > 92%. It has been shown that the diagnosis of VUR by CeVUS has a sensitivity of up to 81% (44/54), and up to 100% in children with a high grade of reflux [17]. Another study showed that the application of CeVUS has a higher sensitivity and specificity for VUR than VUCG [18]. Both in the present study and in related reports from abroad, good agreement was observed between CeVUS and VCUG.

VUR, a childhood urological disorder, is a common primary condition characterized by an abnormal reflux of urine from the bladder into the ureter and even into the pelvis and calyces, with the abnormal physiological structure of the ureter–bladder junction being the most fundamental cause of its formation. In the current study, univariate analysis of urological ultrasound parameters showed that the length of the inner bladder wall segment was closely associated with VUR and that the length of the inner bladder wall segment in the VUR group was significantly shorter than that in the non-VUR group, with statistically significant differences between the groups (*P* < 0.001). Therefore, the timely choice of surgical treatment for children with high-grade VUR can not only avoid the long-term use of antibiotics to prevent drug resistance caused by urinary tract infection but also improve the structural resistance to reflux at the source and eventually achieve a cure for the disease. The current study revealed that the length of the inner segment of the ureteral wall of the bladder in children with VUR was longer after than that before surgery and that the length of the inner segment of the ureteral wall had an AUC value of 1.000, suggesting a high diagnostic value. The ability of two-dimensional ultrasound to display the inner segment of the bladder wall more clearly, combined with the assessment of kidney size, parenchymal thickness, sinus separation, and extent of ureteral dilatation, can effectively provide clinicians with better information.

Nevertheless, CeVUS has certain limitations. Although the existing technology has reduced the destruction of contrast microbubbles and enabled real-time continuous imaging, which makes the posterior bladder tissue imaging subtractive, the acoustic shadow produced by the high concentration of ultrasound contrast agent still obscures the posterior bladder region, impairing the secondary visualization of the posterior bladder wall and reducing the ability of CeVUS to detect class I reflux. This is mainly due to the high molecular weight of the contrast agent, causing microbubbles to float and accumulate in the nondependent part of the bladder (i.e., the anterior portion), resulting in acoustic shadowing [19, 20]. Studies have shown that the ultrasound contrast agent can be diluted via continuous saline bladder perfusion and re-evaluated during the second cycle of examination, a technique which essentially improves the quality of images, resulting in enhanced homogeneity of the bladder cavity and good visualization of the bladder wall [21, 22]. In the current study, a child was re-examined 12 months after surgery, and although no reflux was detected on CeVUS, VCUG revealed grade I reflux. After re-reviewing the ultrasound images, we determined that the ureteral bladder opening was not clearly visualized due to contrast obscuration, which may have affected the diagnostic results. Thus, this aspect should be further explored in the future.

CeVUS of the urethral morphology is less intuitive than VCUG, and the diagnosis of complex urethral anomalies often challenges the proficiency of the sonographer and still requires the combination of CeVUS with VCUG and cystoscopy for diagnostic confirmation when necessary. In some practical situations, the ultrasound contrast agent may be rapidly destroyed due to increased bladder pressure caused by crying and noisy uncooperative children, which might prevent the successful completion of CeVUS. However, this is not a concern for VCUG.

Finally, during the 6-month follow-up after surgery, the current study revealed that some children maintained the same dilated morphology of the pelvic ureter as that observed before surgery despite the decrease in reflux grade and amelioration of symptoms, such as urinary tract infection. Therefore, for such children, evaluation should not be simply based on the morphology of the ureter and pelvis but should be further combined with preoperative dynamic imaging and clinical symptoms for a comprehensive assessment. This warrants further follow-up and observation.

Some limitations of this study include the small number of cases, short follow-up period, and lack of in-depth discussion regarding the limitations of CeVUS and potential solutions. Moreover, we did not have the opportunity to study all urethral anomalies occurring in the general population using CeVUS due to their low prevalence. In our future study, we plan to include a larger sample size, increase the frequency and duration of follow-up, combine CeVUS with three-dimensional ultrasound imaging modalities and urodynamic testing to optimize the diagnostic approach and use better research methods for more in-depth exploration.

## Conclusion

With the increased awareness of radiation protection, radiation dosages to infants and children should be minimized, and radiation-free, efficient, reliable, and safe examinations should be adopted. CeVUS has shown good performance in all aspects of preoperative screening for VUR, follow-up of surgical treatment, and postoperative outcome assessment. Moreover, its application as an effective alternative diagnostic method for VUR is particularly suitable for children requiring multiple postoperative follow-up examinations.

## Data Availability

All data generated or analyzed during this study are available from the corresponding author upon reasonable request.

## Abbreviations

CeVUS: Contrast-enhanced Voiding Urosonography
VUR: Vesicoureteral reflux
VCUG: Voiding cytourethrography
PUUs: Pyelo-ureter units
UTI: Urinary tract infection
